# Systematic Review of Patient Decision Aids for Stroke Prevention Therapy in Atrial Fibrillation Management

**DOI:** 10.31083/j.rcm2310353

**Published:** 2022-10-18

**Authors:** Justin H. Baers, Joel Adekanye, Glen Hazlewood, Jan M. Davies, Jeff K. Caird, Stephen B. Wilton

**Affiliations:** ^1^Department of Community Health Sciences, Cumming School of Medicine, University of Calgary, Calgary, AB T2N 4Z6, Canada; ^2^Department of Medicine, Cumming School of Medicine, University of Calgary, Calgary, AB T2N 2TP, Canada; ^3^Department of Anesthesiology, Perioperative & Pain Medicine, Cumming School of Medicine, University of Calgary, Calgary, AB T2N 2T9, Canada; ^4^Department of Psychology, Faculty of Arts, University of Calgary, Calgary, AB T2N 1N4, Canada; ^5^Libin Cardiovascular Institute, Cumming School of Medicine, University of Calgary, Calgary, AB T2N 4N1, Canada

**Keywords:** atrial fibrillation, patient decision aids, systematic review, stroke prevention

## Abstract

**Background:**

Atrial Fibrillation (AF) is a major cause of stroke. Oral anticoagulation can reduce the risk of AF-associated stroke by 65% but it remains underused. Stroke prevention therapy in patients with AF has been considered a good target for shared decision making with patient decision aids as it is a long-term, preference-sensitive decision with known risk-benefit trade-offs. The aim of this systematic review was to summarize published literature on the effectiveness of patient decision aids on the choice of and adherence to stroke prevention therapy in individuals with AF.

**Methods:**

We conducted a structured literature search for prospective studies evaluating decision aids for AF stroke prevention therapy in adult patients with nonvalvular AF. We included studies that compared those exposed to a decision aid with a control condition for outcomes including choice of therapy, adherence, decisional conflict and patient knowledge. Quantitative meta-analysis was not feasible due to excessive between-study heterogeneity.

**Results:**

Eight studies met inclusion and exclusion criteria. Six studies were randomized clinical trials and two were pre-post comparisons. Of the 8 studies, each evaluated a different decision aid, with only three including all contemporary oral anticoagulant drugs. All decision aids improved AF knowledge compared to baseline or control and decision aids reduced decisional conflict in four of six studies. However, there were inconsistent effects of the studied decision aids on initiation of oral anticoagulation. Adherence to initial stroke prevention therapy choice appeared to benefit from decision aid use in 2 studies that addressed this issue.

**Conclusions:**

Decision aids for stroke prevention increased AF patients’ knowledge and decisional confidence but had variable impacts on choice of and adherence to stroke prevention therapy. The results highlight the need for well-designed decision aids that present patients with all contemporary therapeutic options.

## 1. Introduction 

Atrial Fibrillation (AF) is associated with a 5-fold increase in risk of stroke, accounting for about 15–20% of strokes [1]. This risk can be reduced by approximately 65% with oral anticoagulation (OAC) therapy in appropriately selected patients, at the cost of an increased risk of major bleeding [2]. While all major clinical practice guidelines give the use of OAC a strong recommendation in patients with AF and risk factors for stroke, this therapy remains underused, due in part to misapprehension of the associated risks and benefits among patients and clinicians [3, 4]. For more than two decades, choice of stroke prevention therapy has been considered a good target for shared decision making—and in particular, patient decision aids. This is because choice of stroke prevention therapy is a long-term, non-emergency decision that is preference-sensitive due to the inherent balance of benefits and harms and significant individual variability in underlying stroke risk [5]. The first patient decision aid for AF stroke prevention was tested in 1999, consisting of an audio-booklet with a personalized worksheet [6]. Clinical practice has evolved substantially since that time. Validated clinical prediction scores are now used to select patients most likely to benefit from treatment, and the introduction of direct oral anticoagulants (DOAC), as alternatives to vitamin K antagonists and acetylsalicylic acid (ASA), has increased the complexity of decision-making for patients with AF considering stroke prevention therapy [7, 8, 9].

A Cochrane review focusing on the use of patient decision aids across a broad spectrum of treatment or screening decisions revealed that people exposed to decision aids felt more knowledgeable, better informed and clearer about their personal values, and that they probably had a more active role in the decision-making process and more accurate risk perceptions [10]. A 2017 systematic review reporting on patient decision aids for the choice of stroke prevention therapy in AF management found that decision aid use was associated with patients having increased knowledge, an increased likelihood of making a choice, lower decisional conflict and reduced selection of warfarin [11]. However, it was unclear in that review whether patient decision aid use resulted in increased use of guideline-indicated stroke prevention therapy or improved long-term adherence. New evidence has continued to accrue in this area of study, including recent studies of patient decision aids that incorporated DOAC in the decision matrix. A more recent systematic review by Song *et al*. [12] reported modestly improved uptake of OAC in patients exposed to clinical decision support interventions. However, this study did not differentiate between patient decision aids and physician-focused clinical decision support, which have very different objectives and implementation parameters.

We conducted this updated systematic review to summarize the existing literature reporting on the effectiveness of patient decision aids, as compared with usual care, for stroke prevention decision-making in patients with nonvalvular AF. The primary objective was to determine whether current evidence is sufficient to detect a consistent, favourable effect of the use of patient decision aids for stroke prevention therapy in nonvalvular AF versus usual care on the choice of and/or adherence to stroke prevention therapy. We secondarily sought to determine whether use of these decision aids were associated with measurable differences in process measures related to shared decision making, including decisional conflict and patient knowledge.

## 2. Materials and Methods

### 2.1 Data Sources and Searches

We searched MEDLINE, EMBASE, Cochrane Central Register of Controlled Trials, PUBMED and CINAHL for studies published up to August 2020 that reported on decision aid use within patient populations with nonvalvular AF. Two previously developed Cochrane Review search strategies for decision aids [13] and AF [14] were adapted using the Boolean “and” operator (see **Supplementary Material**). Additional sources were identified through the review of reference lists of all included articles and consultation with AF and shared decision-making experts. Reporting follows the PRISMA guidelines for systematic reviews [15].

### 2.2 Selection Criteria

Studies were eligible for inclusion if they met the following criteria:

(1) The study included adults (≥18 years of age) with nonvalvular AF who were eligible to receive or were receiving stroke prevention therapy (including patients at all stroke risk levels and regardless of comorbidities).

(2) The study involved stroke prevention therapy deliberation with a patient decision aid, defined by the following minimum three-point criteria: explicitly illustrated possible therapy options, specified relevant information about outcomes and therapy and incorporated patients’ values into the decision-making process [13].

(3) The study compared use of the patient decision aid to a control condition.

(4) Reported on at least one of the following outcomes: stroke prevention therapy choice, adherence to stroke prevention therapy choice, decisional conflict, and patient knowledge. Outcome definitions are in Table 1 (Ref. [16, 17]).

**Table 1.** **Reported outcome variables**.

Outcome variable	Definition
Stroke prevention therapy choice	Any reported outcome related to the choice of stroke prevention therapy following intervention (e.g., frequency of therapy selection), discussion of factors related to therapy choice (e.g., why individuals chose a specific therapy) and other relevant information regarding patient preference for therapy.
Adherence to stroke prevention therapy choice	Outcomes related to patient adherence to initial stroke prevention therapy choice ≥3-months post-intervention. Adherence outcomes were based on Dunbar’s [16] three categories of adherence measurements: (1) continuous measurement, like the ratio of medication taken to the medication prescribed over a specific time period; (2) qualitative categories, such as good, acceptable, and poor adherence; and (3) index score based on a variety of behaviors, including adherence to medication and health regimen. This outcome could have also encompassed changes to therapy regimen or non-compliance (e.g., neglecting to take medication).
Decisional conflict	The level of satisfaction individuals face when making decisions that involve risk or challenges to personal life values. Measured using the Decisional Conflict Scale [17]. Acceptable measures of decisional conflict included overall and subscale scores (informed, values clarity, support, uncertainty and effective decision), measured either with a 0–100 or 0–5 scale (lower scores indicated greater decisional confidence and higher scores indicated greater decisional conflict).
Patient knowledge	Any measure of patients’ knowledge (via novel or previously developed scales/questionnaires) about AF, perception of stroke and bleeding risks and/or stroke prevention therapy options.
Additional results	Any additional results related to patient decision aid use (deemed interesting by the reviewers). These results included predictors of stroke prevention therapy choice, patient satisfaction (i.e., level of satisfaction with the decision aid, therapy choice, and/or decision-making process), usability, acceptability, unexpected outcomes, etc.

AF, atrial fibrillation.

All studies that met these criteria, regardless of the specific study design (e.g., observational, pre-post validation, randomized control trial (RCT)) underwent further evaluation. Studies were excluded if the decision aid under assessment was a healthcare provider-only tool, such as a clinical stroke risk calculator. Conference abstracts and other sources of grey literature were also excluded if we were unable to determine if all inclusion criteria were met. No language or publication date restrictions were applied.

### 2.3 Study Identification

After exclusion of duplicate records, two reviewers independently performed eligibility assessments in two stages through a standardized and unblinded approach (i.e., reviewers were aware of the journal publication and author list). The first stage consisted of a title and abstract review based on the inclusion and exclusion criteria, where all eligible articles identified by either reviewer were advanced to the second stage of review. The second stage consisted of a full-text review of all articles that passed the first stage review. Any disagreements were resolved by consensus and after consultation with the senior author.

### 2.4 Data Extraction and Quality Assessment

Two reviewers independently extracted data from the designated list of all eligible studies using a pre-designed data extraction form. The design and reliability of the data extraction form were pilot tested and refined using a random sample of selected studies and according to the efficiency of captured relevant information and consistency in data extraction. Variables extracted included: (1) study characteristics (design, country of origin), sample characteristics (size, stroke risk, baseline stroke prevention therapy, comorbidities); (2) intervention and comparator characteristics; and (3) outcomes (see Table 1). We assessed internal validity in duplicate using the Cochrane Collaboration’s tool for assessing risk of bias for RCTs [18], and the US National Institutes of Health Quality Assessment tool for Observational Cohort and Cross-Sectional Studies, as appropriate [19]. Any disagreements between the reviewers about quality assessment was achieved by consensus including consultation with the senior author. A quantitative meta-analysis was intended if the studies had sufficiently similar variables, were relatively homogenous and permitted valid results to be pooled. However, as the results of this review did not meet these conditions, a quantitative meta-analysis was not justified.

## 3. Results

### 3.1 Search Results

**Fig. 1.**
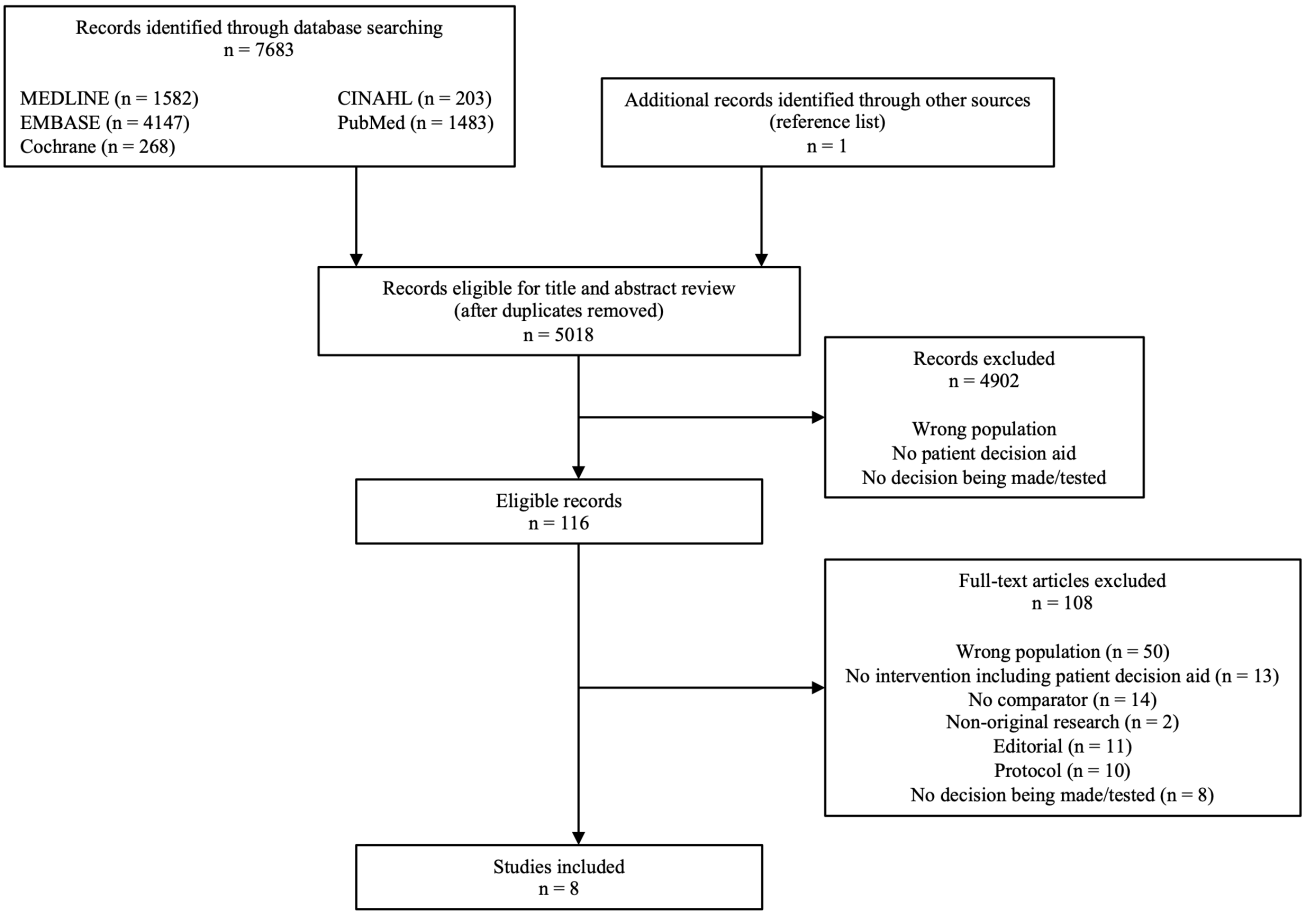
**Flow diagram of literature search and article exclusion**. PDA, patient decision aid.

From the 7683 records identified through the database search, 5018 unique citations were initially identified for title and abstract review (see Fig. 1). Following stage 1 review, 116 manuscripts were selected for full-text review. We excluded 108 articles during the full-text review, for the reasons listed in Fig. 1. The most common reason for exclusion was for incorrect study population (n = 50). Eight studies met all entry criteria and were included in the qualitative review.

### 3.2 Characteristics of Included Studies

Table 2 (Ref. [6, 20, 21, 22, 23, 24, 25, 26]) summarizes the characteristics of the eight studies. These studies were published between 1999 and 2020 and conducted in Brazil (n = 1) [20], Canada (n = 3) [6, 21, 22], China (n = 1) [23], the United Kingdom (n = 1) [24] and the United States (n = 2) [25, 26]. A total of 2153 participants were enrolled across all eight studies. Six of the studies were RCTs, which compared decision aid use to clinical practice guidelines (n = 1) [24] or standard care (n = 5) [6, 21, 23, 25, 26]. The remaining two studies were pre-post studies [20, 22]. Half the decision aids were developed for computer use (n = 4) [22, 24, 25, 26], while two studies tested mobile apps [20, 23] and two studies used audio-booklets [21], one of which was accompanied by a personalized worksheet [6]. Of the eight studies, only Kunneman *et al*. [26] made their decision aid—in its entirety—readily accessible. One study did provide a link to its decision aid, however that link was not functional at the time of attempted access) [21]. Another study specified that the decision aid would be made available upon request [22]. Each report assessed a unique decision aid. The stroke prevention therapy options compared varied across the eight decision aids: all studies included warfarin and compared it to one or more of no therapy, ASA (with or without clopidogrel), and DOACs.

**Table 2.** **Characteristics of included studies**.

Author, year	Country	Study design	Patients (n)	PDA format	Access to entire PDA	Therapeutic options displayed	Administration	Outcomes reported
Man-Son-Hing *et al*. [6] 1999	Canada	RCT (control: standard care)	287	Audio-booklet & personal worksheet	No	ASA vs. warfarin	Self-administered before consultation	Ability to choose SP therapy, adherence at 6 months, knowledge, expectations, decisional conflict, satisfaction in SDM
McAlister *et al*. [21] 2005	Canada	RCT (control: standard care)	434	Audio-booklet	No	ASA vs. warfarin	Self-administered before consultation	Patients receiving SP therapy appropriate to their stroke risk (according to ACCP recommendations), knowledge, expectations, decisional conflict
Thomson *et al*. [24] 2007	United Kingdom	RCT (control: CPG)	109	Computer program	No	warfarin vs. no therapy	During consultation	Decisional conflict, knowledge, decision making preference, SP therapy choice
Fraenkel *et al*. [25] 2012	United States	RCT (control: standard care)	135	Computer program	No	ASA vs. warfarin vs. no therapy	Administered before consultation	Decisional conflict, knowledge, patient-physician communication, change in SP therapy
Guo *et al*. [23] 2017	China	RCT (control: standard care)	209	Mobile app	No	warfarin vs. no therapy (but patient would receive additional DOAC education/counseling if SAMe-${\mathrm{TT}}_{2}$$R_{2}$ score >2)	Self-administered before and after consultation	Knowledge, quality of life, adherence, satisfaction in SDM, usability/feasibility/acceptability
Stephan *et al*. [20] 2018	Brazil	Observational (pre-post validation)	20	Mobile app	No	ASA vs. ASA + clopidogrel vs. warfarin vs. apixaban vs. dabigatran vs. rivaroxaban vs. no therapy	During consultation	Knowledge, decisional conflict, risk perception of OAC
Loewen *et al*. [22] 2019	Canada	Observational (pre-post validation)	37	Computer program	Upon request	Decision 1 (therapeutic class): “ASA” vs. “OAC” vs. “no therapy” vs. “unsure”	Self-administered before consultation	Decisional conflict, knowledge, usability/acceptability, patient preferences, effects on SP therapy choices, participant feedback
						Decision 2 (drug choice; if “OAC” was picked for Decision 1): apixaban vs. dabigatran vs. edoxaban vs. rivaroxaban vs. warfarin		
Kunneman *et al*. [26] 2020	United States of America	RCT (control: standard care)	922	Computer program	Yes	warfarin vs. “DOAC”	During consultation	Quality of communication, knowledge, risk perception, decisional conflict, satisfaction in SDM, decision concordance, duration of encounter, likelihood to recommend encounter

ACCP, American College of Chest Physicians; ASA, acetylsalicylic acid (Aspirin®); CPG, clinical practice guidelines; DOAC, direct oral anticoagulant; HCP, healthcare provider; OAC, oral anticoagulant; PDA, patient decision aid; RCT, randomized control trial; SAMe-${\mathrm{TT}}_{2}$$R_{2}$, warfarin control predictor [Sex, Age <60 years, Medical history, Treatment, Tobacco use, Race]; SDM, shared decision making; SP, stroke prevention.

The process of decision aid delivery was also variable. Most of the decision aids (n = 5) were used by patients outside the clinical visit, either self-administered before (n = 3) [6, 21, 22] and/or after consultation (n = 1) [23] or with the assistance of research staff in preparation for an upcoming consultation (n = 1) [25], while the final three decision aids were designed for co-use by patients and clinicians and administered during the clinical consultation [20, 24, 26].

Studies included patients with AF or at risk of AF. Table 3 (Ref. [6, 20, 21, 22, 23, 24, 25, 26]) summarizes the participant characteristics, which included number of patients, average age, percent female, annual stroke risk, comorbidities and stoke prevention therapy at baseline. The sample size of the eight studies ranged from 20 to 922 patients [20, 26]. The majority of patients were at least 70 years of age. The proportion of females varied, ranging from 1% to 57% of the sample populations [22, 25]. Patients typically had a high annual risk of stroke. Seven of the eight studies predominantly included patients who had previous exposure to OAC (predominately warfarin or unspecified) [6, 20, 21, 22, 24, 25, 26]; the other study did not report stroke prevention therapy at baseline [23].

**Table 3.** **Participant characteristics**.

Author, year	Participants (n)	Mean age (years)	Female (%)	Annual stroke risk (%)	Comorbidities (%)	Stroke prevention therapy at baseline (%)
Man-Son-Hing *et al*. [6] 1999	287 AF	66	24	Not reported	Hypertension (41)	ASA (43), Warfarin (*ever taken*; 26)
McAlister *et al*. [21] 2005	434 AF	72	39	Low (8), moderate-low (9), moderate-high (3), high (39), very high (41)	CAD (32), diabetes (18), heart failure (20), hypertension (56), prior stroke (22)	ASA (9), warfarin (79), ASA + warfarin (10), no therapy (2)
Thomson *et al*. [24] 2007	109 AF	73	44	Average: low-moderate (annual stroke risk: 2.16%)	Not reported	ASA (23), warfarin (71)
Fraenkel *et al*. [25] 2012	135 AF	Majority ≥75	1	Low (4), moderate (24), high (72)	Diabetes (28), heart failure (26), hypertension (87), prior stroke (8)	ASA (8), warfarin (73), ASA + warfarin (19)
Guo *et al*. [23] 2017	209 AF	69	44	Average: high (${\mathrm{CHA}}_{2}$${\mathrm{DS}}_{2}$-VASc score = 2.65)	CAD (44), diabetes (17), heart failure (15), hypertension (58), hypertrophic cardiomyopathy (3), liver dysfunction (2), PAD (5), prior stroke (9), renal dysfunction (6)	Not reported
Stephan *et al*. [20] 2018	20 AF	68	40	Low (3), moderate (10), high (87)	Alcohol abuse (3), cardiovascular disease (23), diabetes (30), heart failure (30), history of bleeding (17), hypertension (80), non-ASA NSAIDs (27), prior stroke (17), pulmonary disease (17), renal dysfunction (7), SBP >160 mmHg (10), smoking (10)	Unspecified OAC (67); no therapy (33)
Loewen *et al*. [22] 2019	37 AF & at risk of AF	71	57	Average: high (${\mathrm{CHA}}_{2}$${\mathrm{DS}}_{2}$-VASc score = 2.38)	Diabetes (5), heart failure (21), history of bleeding (21), hypertension (40), labile INR (23), liver dysfunction (5), myocardial infarction (16), prior stroke (8), renal dysfunction (13), SBP >160 mmHg (18)	ASA (27), warfarin (14), apixaban (16), dabigatran (0), rivaroxaban (19), edoxaban (0), no therapy (27)
Kunneman *et al*. [26] 2020	922 AF	71	37	Low (0), moderate (8), high (92)	Not reported	Unspecified OAC (79), no therapy (21)

AF, atrial fibrillation; ASA, acetylsalicylic acid (Aspirin®); CAD, coronary artery disease; INR, international normalized number; PAD, peripheral arterial disease; PDA, patient decision aid; SBP, systolic blood pressure.

### 3.3 Outcomes

The outcomes assessed in each study are characterized in Table 4 (Ref. [6, 20, 21, 22, 23, 24, 25, 26]), which include stroke prevention therapy choice, adherence to stroke prevention therapy choice, decisional conflict, patients’ knowledge and additional results.

**Table 4.** **Outcomes assessed in included studies examining patient decision aids for stroke prevention therapy in atrial fibrillation management**.

Author, year	Stroke prevention therapy choice	Adherence to stroke prevention therapy choice	Decisional conflict (Overall & Subscales)	Patient knowledge	Additional results
Man-Son-Hing *et al*. [6] 1999	PDA group more likely to make a definitive choice about SP therapy (ASA vs. warfarin) following consultation compared to standard care (99% vs. 94%, *p* = 0.02)	No difference in adherence to initial SP therapy choice at 6 months (6 patients changed their SP therapy plans in PDA group vs. 9 patients in standard care, *p* = 0.44)	No difference in overall decisional conflict (*p* = 0.14), but patients using PDA felt more informed compared to standard care (*p *< 0.05)	Compared to standard care: PDA improved knowledge about AF and SP therapy options; higher percentage of patients in PDA group gave accurate estimates of their stroke and bleeding risks when taking ASA and warfarin	No difference in satisfaction with DM process (*p* = 0.1); previous warfarin use was an independent predictor of choosing warfarin as initial SP therapy (*p* = 0.04)
McAlister *et al*. [21] 2005	Not reported	Not reported	PDA lowered overall decisional conflict, and patients using PDA felt more certain (*p* = 0.02), more informed (*p *< 0.001), and clearer about personal values (*p* = 0.04) compared to standard care	PDA group more accurate in their estimates of potential benefits and risks of SP therapy (*p *< 0.05)	12% absolute improvement in number of individuals with AF receiving appropriate SP therapy in PDA group vs. standard care at 3 months (*p* = 0.03) but no difference seen at 12 months (based on guideline recommendations)
Thomson *et al*. [24] 2007	PDA group less likely to make a definitive choice regarding SP therapy (warfarin vs. no therapy) compared to CPG (OR = 0.33); patients not already on warfarin less likely to start warfarin in PDA group (OR = 0.01)	Not reported	PDA lowered overall decisional conflict compared to CPG (*p* = 0.036); PDA patients felt more informed and clearer about personal values for risks and benefits of options (*p *< 0.05)	No difference in knowledge between PDA and CPG groups	No difference in number of HCP consultations and hospitalizations at 3 months following initial consultation between groups (*p *> 0.05)
Fraenkel *et al*. [25] 2012	No change in SP therapy choice in PDA or standard care groups post-30 days; 5 patients on warfarin in PDA group expressed ASA to be a better SP therapy choice for them, but HCP convinced them otherwise	Not reported	Difference in overall decisional conflict not reported, but patients using PDA felt more informed (*p* = 0.011) and clearer about personal values for risks and benefits of options compared to standard care (*p *< 0.001)	Compared to standard care: PDA improved knowledge about SP therapy options and side effects; PDA group more accurate in their stroke and bleeding risks	Compared to standard care, PDA increased the number of discussions about stroke and bleeding risks with HCP (*p *< 0.0001)
Guo *et al*. [23] 2017	PDA group more likely to choose DOAC compared to standard care (*p *< 0.001)	Greater adherence levels in PDA group at 1- and 3-months compared to standard care (*p *< 0.05)	Not reported	PDA improved knowledge about AF compared to standard care (*p <* 0.05)	Compared to standard care, PDA increased QoL scores and reduced anxiety and depression (*p *< 0.05); >90% of patients found PDA easy, user-friendly and helpful
Stephan *et al*. [20] 2018	Not reported	Not reported	Overall decisional conflict was low after PDA use (DCS: 11 ± 16/100); decisional conflict was not measured at baseline	Knowledge about AF was greater after PDA use compared to baseline (*p *< 0.001), but there was no difference in accuracy of risk perception	Not reported
Loewen *et al*. [22] 2019	Among Individuals with AF, 20% chose a SP therapy from a therapeutic class (ASA vs. OAC vs. no therapy) different from that currently prescribed to them; 60% chose a different drug than that currently prescribed to them	Not reported	Overall decisional conflict (MD, –21.1; 95% CI, –31.7 to –21.2) and its subscales were lower after PDA use compared to baseline	Knowledge about AF was greater after PDA use compared to baseline (*p* = 0.02)	89% of patients completed PDA in a single session; 76% of patients felt individualized therapy attribute ranking was congruent with their values; PDA well accepted; SUS score = 61/100; no negative consequence of using PDA identified
Kunneman *et al*. [26] 2020	Decision concordance high in both PDA and standard care groups	Not reported	No difference in decisional conflict between PDA group and standard care (for overall decisional conflict and its subscales)	No difference in knowledge about AF and SP therapy options (aRR, 1.01; 95% CI, 1.0 to 1.02) and risk perception between (strict aRR, 1.4; 95% CI, 0.8 to 2.2 and liberal aRR, 1.3; 95 CI%, 0.8 to 1.8) PDA group and standard care	Communication quality reported high in both PDA and standard care groups; both PDA and standard care groups recommended their approach used; clinicians more satisfied after PDA use compared to standard care (aRR, 1.49; 95% CI, 1.42 to 1.53); no difference in encounter duration (approx. mean duration: 31–32 min, aMD, 1.1.; 95% CI, –0.3 to 2.5 min)

aMD, adjusted between-arm difference; aRR, adjusted relative risk; ASA, acetylsalicylic acid (Aspirin®); CPG, clinical practice guidelines; CI, confidence interval; DCS, Decisional Conflict Scale; DM, decision-making; DOAC, direct oral anticoagulant; HCP, healthcare providers; MD, mean difference; OAC, oral anticoagulant; OR, odds ratio; PDA, patient decision aid; QoL, quality of life; SP, stroke prevention; SUS, System Usability Scale.

### 3.4 Risk of Bias Assessment

**Fig. 2.**
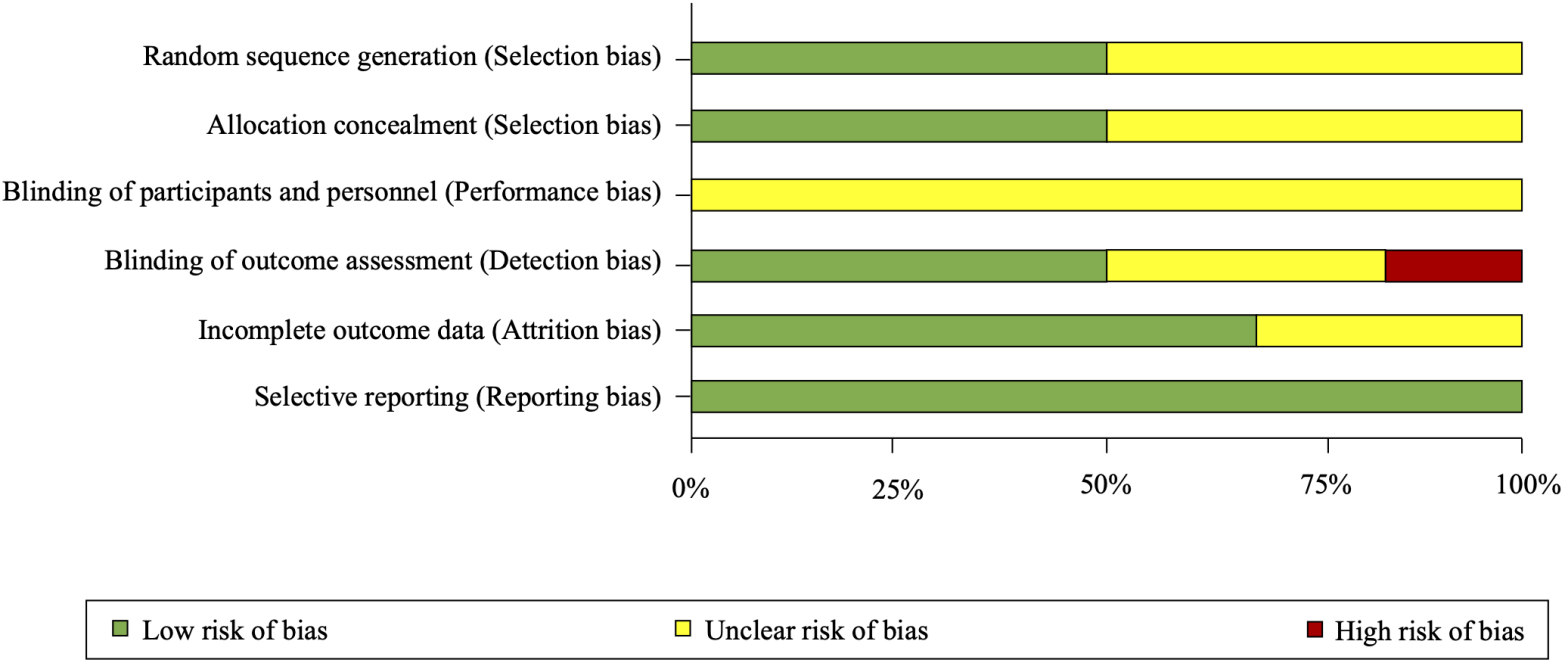
**Risk of bias summary as percentages across all included randomized trials**.

**Fig. 3.**
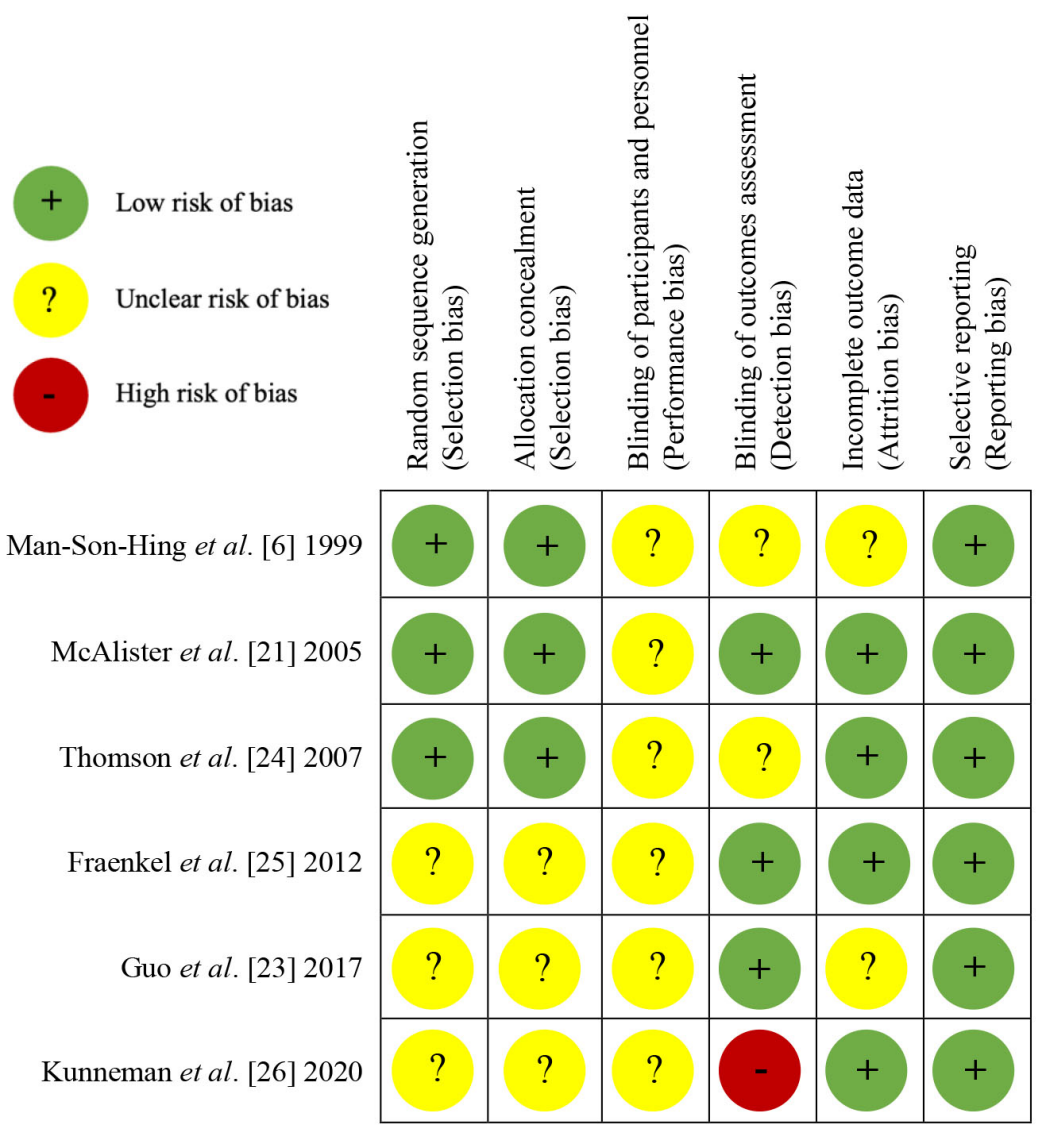
**Risk of bias summary for each included randomized trial**. Summary of risk of bias assessment of included randomized trials conducted using the Cochrane Collaboration’s Risk of Bias tool [18]. Green circles with a ‘+’ indicate low risk of bias, yellow circles with a ‘?’ indicate unclear risk of bias and red circles with a ‘-’ indicate high risk of bias.

Overall, the six RCTs were rated at low or uncertain risk of bias (Fig. 2; Fig. 3, Ref. [18]). See **Supplementary Table 1**, for expanded details on risk of bias in each included trial.

The quality of the two observational studies was rated as “fair” (See **Supplementary Table 2** for further details on the rationale for these ratings).

### 3.5 Stroke Prevention Therapy Choice

The second column in Table 4 summarizes the results of reported stroke prevention therapy choice. Six of the eight studies used this outcome. Man-Son-Hing *et al*. [6] found that individuals with AF who used their decision aid were more likely to make definite stroke prevention therapy choices (99% vs. 94%, *p* = 0.02). Conversely, Thomson *et al*. [24] found that individuals with AF who used their decision aid were less likely to make a decision to start or continue warfarin, a finding that was entirely due to a marked difference in the group of patients not already taking warfarin (25% vs. 94%, relative risk 0.27, 95% CI, 0.11 to 0.63). Fraenkel *et al*. [25] noted no change in stroke prevention therapy choice following both decision aid use or standard care consultations. However, following consultation with their decision aid, five patients (7.6% of the intervention group) indicated a preference for changing their current warfarin therapy regimen to ASA, but were convinced otherwise by physicians with a strong preference for warfarin therapy (four of the five cases), or by a medical trainee who felt uncomfortable allowing a transition in therapy. Guo *et al*. [23] found patients in their decision aid group were more likely to choose a DOAC compared to their standard care group. Loewen *et al*. [22] reported that, by using their decision aid, 20% of their individuals with AF chose a stroke prevention therapy from a therapeutic class (antiplatelet vs. OAC vs. no therapy) different from that currently prescribed to them and 60% of individuals with AF chose a different drug than the one currently prescribed to them. Lastly, Kunneman *et al*.’s [26] study showed decision concordance, which is the therapeutic alliance and negotiation reached between patients and their healthcare providers, to be high in both their decision aid and standard care groups.

### 3.6 Adherence to Stroke Prevention Therapy Choice

Only two of the eight studies reported adherence to initial stroke prevention therapy choice (≥3-months post-intervention), as shown in Table 4 [6, 23]. Guo *et al*. [23] assessed patients’ adherence at three months using scores from a 3-item Adherence Estimator, whereas Man-Son-Hing *et al*. [6] assessed patients’ adherence at six months using telephone follow-up inquiring about current therapy and reasons for any change from the original decision. Guo *et al*. [23] found patients’ adherence levels to be greater with the use of their decision aid compared to standard care. However, Man-Son-Hing *et al*. [6] found patients’ adherence to be similar for their decision aid and standard care groups.

### 3.7 Decisional Conflict

Seven studies reported decisional conflict, as measured by the validated Decisional Conflict Scale (DCS) [17]. However, as shown in the fourth column in Table 4, reporting of the DCS varied across studies. The variability in the application of this outcome precluded a valid quantitative meta-analysis. Six of those seven studies reported their overall DCS scores, either in comparison to standard care [6, 21, 26] or before decision aid use [22], as a mean difference between decision aid use and clinical practice guidelines [24], or in one instance, with no comparator at all [20]. Four of the seven studies measured and clearly reported all five of their decisional conflict subscale scores (i.e., effective, informed, support, uncertainty and values clarity) [6, 21, 22, 26], two of the seven studies only measured and reported some of the decisional conflict subscales (Fraenkel *et al*. [25]: informed and values clarity; and Loewen *et al*. [22]: informed, support, uncertainty and values clarity) and one study stated they measured the following three subscales but did not report their individual scores: uncertainty, values clarity and support [20]. In respect to overall decisional conflict scale scores: two studies found that the decision aid led statistically significant but small magnitude improvements in decisional confidence compared to control groups [21, 24]; one study indicated patients who used a decision aid had greater decisional confidence after decision aid use compared to baseline [22]; one study reported low decisional conflict after decision aid use but did not measure it before use [20]; and two studies reported no difference in decisional confidence between the decision aid and standard care groups [6, 26]. In terms of the DCS subscale scores and the respective studies that reported them, patients who used a decision aid felt more informed (n = 5/6; compared to control or baseline) [6, 21, 22, 24, 25], better supported (n = 1/5; compared to baseline) [22], more certain (n = 2/5; compared to control or baseline) [21, 22] and clearer about personal values (n = 4/6; compared to control or baseline) [21, 22, 24, 25].

### 3.8 Patient Knowledge

All eight studies reported outcomes for patients’ knowledge (Table 4). Each study reported using a different knowledge assessment tool, evaluating one or more of: AF knowledge (n = 5); accuracy of risk perception for both stroke and bleeding (n = 4); and understanding of stroke prevention therapy options, including benefits, risks and side effects (n = 4). Decision aid use improved general AF knowledge (n = 4/5, compared to control or baseline) [6, 20, 22, 23], accuracy of risk perception (n = 3/4, compared to control or baseline) [6, 21, 25], and understanding of stroke prevention therapy options (n = 2/4, compared to control) [6, 25].

### 3.9 Additional Results

The sixth column in Table 4 summarizes the additional results reported by each study. Compared to standard care, decision aid use improved the number of individuals with AF receiving appropriate stroke prevention therapy three months after initial consultation with the patient about the use of a decision aid [21]; increased the number of discussions about stroke and bleeding risks during consultations [25]; and was associated with better quality of life scores, reduced anxiety and depression, as well as greater healthcare providers’ satisfaction [23, 26]. Additionally, patients reported high satisfaction and communication quality, and would recommend the use of decision aids, although patients’ satisfaction, communication quality and recommendation were also high in standard care groups [6, 26]. Decision aid use was not associated with a difference in the number of subsequent clinic visits within three months in one study [24], nor with the duration of patients’ visits compared to control in another study [26]. Lastly, two of the eight studies reported assessing the acceptability and usability of their decision aids, and found that they could be used independently by and were acceptable to patients [22, 23].

## 4. Discussion 

This systematic review and narrative synthesis included eight articles that examined the effects of patient decision aids on individuals with or at risk of AF, in choosing stroke prevention therapy. Due to the very significant heterogeneity in the design and implementation of the individual patient decision aids, as well as the interventions compared in each study, a quantitative meta-analysis was not performed, in accordance with best practices in systematic reviews [27, 28]. Therefore, a pooled estimate of the effects of the studied decision aids on our primary outcomes of stroke prevention therapy choice and adherence is unavailable [6, 22, 23, 24, 25, 26]. However, it was apparent that decision aid use increased patients’ knowledge and decisional confidence. We found decision aid use improved general AF knowledge in 80% of the studies [6, 20, 22, 23], accuracy of risk perception in 75% of the studies [6, 21, 25], and understanding of stroke prevention therapy options in 50% of the studies [6, 25].

The strengths of this systematic review include our comprehensive search strategy, inclusion of all relevant study designs and a rigorous quality assessment. We summarized the design characteristics, implementation methods and results of the decision aids trialed in the included studies. While significant between-study variability precludes making definitive statements about the relative merits of the various included design features, we believe this work provides a valuable reference for researchers working in this field.

Despite over 20 years of research, it remains unclear whether use of patient decision aids leads more patients with AF to select a guideline-recommended stroke prevention therapy or encourages better long-term adherence. This review identified several potential sources of this uncertainty, including variability in decision aid tool design, delivery, and evaluation metrics, which limit opportunities for quantitative meta-analysis. As such, important questions relevant to researchers designing decision aids and to clinicians considering their use in practise, remain unanswered. These include optimal tool design (paper-, computer-, or app-based) delivery format (pre-encounter or in-visit), and the role of repeated interventions. Furthermore, most studies included patients with high baseline exposure to AF stroke prevention decision-making: 99% of the patient population were already familiar with stroke prevention therapy through their previous experiences and 73% of patients were already taking an OAC/DOAC at baseline [6, 22, 24]. As a result, this previous experience could have biased patients towards their current stroke prevention therapy regimen because the patients were reported as generally satisfied with their current therapy regimen. This result could relate to patients potentially desiring familiarity with their ongoing therapy or having no difficulty in deciding on their best course of action, for example, if they already had high levels of knowledge and decisional confidence at baseline. This decision-making bias, known as anchoring or status quo bias, reduces the potential contribution of decision aids in AF management for patients who already have an individualized care plan [29]. Future studies should consider recruitment of larger proportions of newly-diagnosed or treatment-naïve AF patients.

Consistent with more general reviews of patient decision aids for various disease conditions and purposes, our results show decision aid use is associated with improved risk perception and decisional confidence [10]. All included studies found that decision aid use was associated with improved AF knowledge [6, 20, 22, 23] and/or the understanding of stroke prevention therapy options [6, 25]. The fact that improvements in knowledge do not necessarily translate to behaviour changes is well-known in behavioural science and is sometimes called the ‘Knowledge-Attitude-Behaviour Gap’. These results emphasize that in order to change behaviour and clinical outcomes, even the most effective patient decision aids will need to be carefully implemented and serve to augment rather than replace the clinician-patient relationship.

Interestingly, one study found that five patients preferred ASA rather than their current warfarin regimen after the use of a decision aid, but were convinced by their respective healthcare providers to continue taking warfarin [25]. This example emphasizes issues related to the use of patient decision aids within clinical practice, particularly if the providers views, references, and /or beliefs about the evidence do not align with the information presented in the decision aid. Some healthcare providers may harbour a belief that patients do not—and should not—have a choice about their therapeutic options. This represents a barrier to decision aid use in clinical care [30]. Thus, expanded efforts are needed with decision aid development that includes healthcare providers in the development process.

We also found some of the evaluated patient decision aids to be outdated. Only three studies [20, 22, 26] in this review incorporated a DOAC as a stroke prevention therapy option. Future decision aids should ensure all contemporary therapeutic options, including non-pharmacological options such as left atrial appendage closure, are included. The inclusion of these therapies will increase the complexity of therapy deliberation, further emphasizing the need for decision aids, and potentially for ancillary decision support measures such as decision coaching [31]. In addition, we found most of the decision aids to be publicly unavailable. This is likely due to the lack of resources necessary to update decision aids to include all contemporary therapeutic options, as well as to maintain them in the public domain.

Lastly, while it appears that decision aid use is associated with greater knowledge and decisional confidence, this does not necessarily mean that patient decision aids are designed well. Only two studies reported designing their decision aids according to the International Patient Decision Aid Standards (IPDAS) criteria (albeit several of the decision aids were designed before the first IPDAS criteria were published) [22, 25]. Future decision aid development should therefore consider conforming their design to the IPDAS criteria as a way to optimize their development. Moreover, only one study reported using the results of a formative assessment to refine their decision aid’s development, but that study provided no detail on and reported no outcomes from the testing and participant feedback [22]. Additionally, two of the eight studies reported conducting summative assessments of their decision aids [22, 23]. Considering that decision aid use requires engagement from both patients and healthcare providers, obtaining their feedback on usability, content and acceptability and incorporating their suggestions directly into the design process will optimize decision aid development [32]. This will also increase usability by ensuring decision aids are not perceived as time-consuming and are relevant to individuals’ health concerns.

## 5. Limitations

This systematic review has several limitations. First, the total number of participants was relatively small. This small number may have influenced the generalizability of the results and makes determining any causal relationships with the use of patient decision aids more difficult. Moreover, one study included some patients (n = 12/37; 32%) that were at risk of, but did not have, AF [22]. Second, the small number of studies and the heterogeneity in study design, interventions and outcome reporting precluded meaningful quantitative meta-analysis and formal assessment of publication bias. Future research should therefore consider establishing a core set of well-defined outcome measures related to decision aid use, which researchers can routinely use. Future evaluation studies of decision aid use should also consider following the Standards for Universal Reporting of Patient Decision Aid Evaluation Studies (SUNDAE) standards to improve the quality of their publications (which one study did) [22, 33]. Lastly, none of the studies evaluated the efficacy of their patient decision aid’s use on clinical outcomes such as stroke and bleeding.

## 6. Conclusions

In this systematic review we found that current evidence for the use of patient decision aids to influence initial stroke prevention therapy choice or longer-term adherence with stroke prevention therapy for patients with non-valvular AF is inconclusive. The decision aids we studied did reduce decisional conflict and increase patients’ knowledge. These findings highlight the need for well-designed decision aids that present patients with all contemporary therapeutic options. Future research is also needed to evaluate stroke prevention therapy choice in individuals who were recently diagnosed with AF, subsequent long-term adherence to this treatment and attention to barriers to decision aid implementation.

## Data Availability

The datasets supporting the analysis and conclusions of this systematic review can be found in Fig. 1, Tables 1,2,3,4, Supplementary Information, **Tables 1,2**, **Figs. 1,2**.

## Protocol Registration

None. Protocol can be accessed by contacting corresponding author.

## Abbreviations

ACCP, American College of Chest Physicians; AF, Atrial Fibrillation; aMD, adjusted between arm difference; aRR, adjusted relative risk; ASA, Acetylsalicylic acid; CAD, coronary artert disease; CI, confidence interval; CPG, clinical practice guidelines; DCS, Decisional Conflict Scale; DM, decision-making; DOAC, Direct oral anticoagulant (non-vitamin K antagonist oral anticoagulant); HCP, health care provider; INR: international normalized ratio; IPDAS, International Patient Decision Aid Standards; MD, mean difference; OAC, Oral anticoagulant; OR, odds ratio; PAD, peripheral arterial disease; PDA, patient decision aid; QoL; quality of life; SAMe-TT2R2, warfarin control predictor [Sex, Age <60 years, Medical history, Treatment, Tobacco use, Race]; SBP, systolic blood pressure; SDM, shared decision making; SP, stroke prevention; SUNDAE, Standards for Universal Re- porting of Patient Decision Aid Evaluation Studies; SUS, System Usability Scale; RCT, Randomized control trial.

## Supplementary Material

Supplementary material associated with this article can be found, in the online version, at https://doi.org/10.31083/j.rcm2310353.
