# Supplementary material for: Systematic Review of Patient Decision Aids for Stroke Prevention Therapy in Atrial Fibrillation Management
Source: Rev Cardiovasc Med. 2022 Oct 18;23(10):353. doi: 10.31083/j.rcm2310353 (PMC11267376; doi:10.31083/j.rcm2310353)
Supplement: Supplementary file 1 [file 2153-8174-23-10-353-s1.zip › 2153-8174-23-10-353-s1/v0-Supplementary material.docx]

Supplementary Information for Baers *et al*. “A Systematic Review of Patient Decision Aids for Stroke Prevention Therapy in Atrial Fibrillation Management”

**Decision Aid Strategy Search**

Cochrane Review search strategy for *decision aids:[6]*

**MEDLINE**

1. decision support techniques/
2. decision support systems clinical/
3. decision trees/
4. (decision making or choice behavior).mp. and informed consent.sh.
5. ((decision* or decid*) adj4 (support* or aid* or tool* or instrument* or technolog* or technique* or system* or program* or algorithm* or process* or method* or intervention* or material*)).tw.
6. (decision adj (board* or guide* or counseling)).tw.
7. ((risk communication or risk assessment or risk information) adj4 (tool* or method*)).tw.
8. decision-making computer assisted/
9. (computer* adj2 decision making).tw.
10. interactive health communication*.tw.
11. (interactive adj (internet or online or graphic* or booklet*)).tw.
12. (interacti* adj4 tool*).tw.
13. ((interactiv* or evidence based) adj3 (risk information or risk communication or risk presentation or risk graphic*)).tw.
14. shared decision making.tw.
15. (informed adj (choice* or decision*)).tw.
16. adaptive conjoint analys#s.tw.
17. or/1-16

**EMBASE**

1. decision support system/
2. patient decision making/
3. decision aid/
4. “decision tree”/
5. decision making.hw,kw,tw. and informed consent.hw,kw.
6. ((decision* or decid*) adj4 (support* or aid* or tool* or instrument* or technolog* or technique* or system* or program* or algorithm* or process* or method* or intervention* or material*)).tw,kw.
7. (decision adj (board* or guide* or counseling)).tw,kw.
8. ((risk communication or risk assessment or risk information) adj4 (tool* or method*)).tw,kw.
9. (computer* adj2 decision making).tw,kw.
10. interactive health communication*.tw,kw.
11. (interactive adj (internet or online or graphic* or booklet*)).tw,kw.
12. (interacti* adj4 tool*).tw,kw.
13. ((interactiv* or evidence based) adj3 (risk information or risk communication or risk presentation or risk graphic*)).tw,kw.
14. shared decision making.tw,kw.
15. (informed adj (choice* or decision*)).tw,kw.
16. adaptive conjoint analys#s.tw,kw.
17. or/1-16

**Cochrane Central Register of Controlled Trials**

The Cochrane Library was searched with the same search strategy as MEDLINE, as shown above.

**CINAHL**

1. exp Decision Making/
2. information seeking behavior/
3. Help Seeking Behavior/
4. (choic$ or preference$).tw.
5. decision$.tw.
6. Educational Technology/
7. or/1-6
8. exp Health Behavior/
9. consumer participation/
10. exp Health Education/
11. health knowledge/ or exp professional knowledge/
12. exp Consent/
13. informed consent.tw.
14. patient.tw,hw.
15. consumer.tw,sh.
16. or/8-15
17. 7 and 16

**Atrial Fibrillation Search Strategy**

Cochrane Review search strategy for *atrial fibrillation [7]:*

**MEDLINE**

1. Atrial Fibrillation/
2. Atrial Flutter/
3. ((atrial or atrium or auricular) adj3 (fibrillat$ or flutter$)).tw.
4. or/1-3

**EMBASE**

1. Heart Atrium Fibrillation/
2. Heart Atrium Flutter/
3. ((atrial or atrium or auricular) adj3 (fibrillat$ or flutter$)).tw.
4. or/1-3

**Cochrane Central Register of Controlled Trials**

The Cochrane Library was searched with the same search strategy as MEDLINE, as seen above.

**CINAHL**

1. Atrial Fibrillation/
2. Atrial Flutter/
3. ((atrial or atrium or auricular) adj3 (fibrillat$ or flutter$)).tw.
4. or/1-3

######

Supplementary Table 1. Risk of Bias Designation Rationale for Quality Assessment of Included Studies.

| **Author, Year** | **Random Sequence Generation** (s*election bias*) | **Allocation Concealment** (s*election bias*) | **Blinding of Participants and Personnel**  (p*erformance bias*) | **Blinding of Outcome Assessment**  (d*etection bias*) | **Incomplete Outcome Data**  (a*ttrition bias*) | **Selective Reporting**  (r*eporting bias*) |
| --- | --- | --- | --- | --- | --- | --- |
| Man-Son-Hing *et al*. 1999[3] | Low Risk  (“computer-generated scheme”; pg. 738) | Low Risk  (“administered from a central location to block the sequence from previewing”; pg. 738) | Unclear Risk  (“physicians may have provided DA information to patients receiving standard care”; pg. 743) | Unclear Risk  (not stated) | Unclear Risk  (attrition mentioned in flow diagram, but no reason given, baseline data not provided; pg. 740) | Low Risk  (transparent reporting of results) |
| McAlister *et al*. 2005[14] | Low Risk  (“computer-generated sequence”; pg. 497) | Low Risk  (“done centrally to preserve allocation concealment”; pg. 497) | Unclear Risk  (“patients and their physicians were not blinded to group allocation”; pg. 497) | Low Risk  (“although patients and their physicians were not blinded to group allocation, outcome assessors were”; pg. 497) | Low Risk  (Results & flow diagram; pg. 498) | Low Risk  (“DAAFI Trial protocol … has been previously published”; pg. 496) |
| Thomson *et al*. 2007[17] | Low Risk  (“electronically-generated random permuted blocks via a web-based randomisation”; pg. 217) | Low Risk  (“service provided by the Centre for Health Services Research”; pg. 217) | Unclear Risk  (Physicians were blinded, but unclear whether patients were) | Unclear Risk  (not stated) | Low Risk  (Flow diagram; pg. 218) | Low Risk  (“trial is registered … ISRCTN24808514”; pg. 222) |
| Fraenkel *et al*. 2012[18] | Unclear Risk  (“clustered randomized controlled trial” using only two institutions; pg. 1434) | Unclear Risk  (not stated) | Unclear Risk  (not stated) | Low Risk  (“research assistant blinded to intervention assignment”, interviewer blinded to the participant’s group assignment”; pg. 4) | Low Risk  (flow diagram; pg. 10) | Low Risk  (DA protocol has been published; gov: NCT00829478) |
| Guo *et al*. 2017[16] | Unclear Risk  (“a cluster randomized design based in 2 hospitals”; pg. 1398) | Unclear Risk  (not stated) | Unclear Risk  (not stated) | Low Risk  (“Data input into analysis was performed by 2 individuals, who were blinded for the intervention”; pg. 1390) | Unclear Risk  (not stated) | Low Risk  (“registered in the Chinese Clinical Trial Registry, International Clinical Trials Registry Platform of the World Health Organization [ChiCTR-IOR-17010436]”; pg. 1390) |
| Kunneman *et al*. 2020[19] | Unclear Risk  (encounters were randomized using a randomization algorithm and analyzed using the intention-to-treat principle, but clinicians participated in both arms—meaning “bias could have been introduced when enrolled clinicians chose not to enroll an eligible patient encounter into the clinical trial”; pg. E8) | Unclear Risk  (allocation concealment mentioned but no detail provided; pg. E8) | Unclear Risk  (“bias may have affected the unblinded assessment of recorded encounters and the scoring of those encounters”; pg. E8) | High Risk  (Reviewers were not blinded to intervention assignment; E3) | Low Risk  (Flow diagram; pg. E6) | Low Risk  (“the study protocol for the clinical trial was published previously”; pg. E2) |

Supplementary Table 2. Rationale for Quality Assessment of Observational Studies.

| **Components** | **Stephan *et al.* 2018** | **Loewen *et al.* 2019** |
| --- | --- | --- |
| *1. Was the research question or objective in this paper clearly stated?* | Yes (“Objective”; pg. 7) | Yes (“Objective”; pg. 666) |
| *2. Was the study population clearly specified and defined?* | Yes (“The study population comprised”; pg. 9) | Yes (“defined as”; pg. 667) |
| *3. Was the participation rate of eligible persons at least 50%?* | Not reported | Not reported |
| *4. Were all the subjects selected or recruited from the same or similar populations (including the same time period)? Were inclusion and exclusion criteria for being in the study prespecified and applied uniformly to all participants?* | Yes (“in April and May 2016”; pg. 9) | Yes (“Enrollment occurred between June 2016 and February 2017”; pg. 669) |
| *5. Was a sample size justification, power description, or variance and effect estimates provided?* | Yes (“18 patients were required for a 5% alpha error and a beta error of 90%”; pg. 9) | No |
| *6. For the analyses in this paper, were the exposure(s) of interest measured prior to the outcome(s) being measured?* | Yes (primary outcome: “analyzed … before and after the interaction with the app”; pg. 9) | Yes (“performed a before-and-after observational study”; pg. 67) |
| *7. Was the timeframe sufficient so that one could reasonably expect to see an association between exposure and outcome if it existed?* | Cannot determine | Cannot determine |
| *8. For exposures that can vary in amount or level, did the study examine different levels of the exposure as related to the outcome (e.g., categories of exposure, or exposure measured as continuous variable)?* | Not applicable | Not applicable |
| *9. Were the exposure measures (independent variables) clearly defined, valid, reliable, and implemented consistently across all study participants?* | Yes | Yes |
| *10. Was the exposure(s) assessed more than once over time?* | No (“analyzed … before and after the interaction with the app”; pg. 9) | No (“performed a before-and-after observational study”; pg. 67) |
| *11. Were the outcome measures (dependent variables) clearly defined, valid, reliable, and implemented consistently across all study participants?* | Yes | Yes |
| *12. Were the outcome assessors blinded to the exposure status of participants?* | Not reported | Not reported |
| *13. Was loss to follow-up after baseline 20% or less?* | Not reported | Not reported |
| *14. Were key potential confounding variables measured and adjusted statistically for their impact on the relationship between exposure(s) and outcome(s)?* | No | No |
| **Quality Rating** | Fair | Fair |
| *Additional Comments (If Poor, please state why):* |  |  |
